# SEP-class genes in *Prunus mume* and their likely role in floral organ development

**DOI:** 10.1186/s12870-016-0954-6

**Published:** 2017-01-13

**Authors:** Yuzhen Zhou, Zongda Xu, Xue Yong, Sagheer Ahmad, Weiru Yang, Tangren Cheng, Jia Wang, Qixiang Zhang

**Affiliations:** Beijing Key Laboratory of Ornamental Plants Germplasm Innovation & Molecular Breeding, National Engineering Research Center for Floriculture, Beijing Laboratory of Urban and Rural Ecological Environment, Key Laboratory of Genetics and Breeding in Forest Trees and Ornamental Plants of Ministry of Education, School of Landscape Architecture, Beijing Forestry University, Beijing, 100083 China

**Keywords:** SEP genes, *Prunus mume*, Floral organ development, Expression analysis, Yeast two-hybrid assay

## Abstract

**Background:**

Flower phylogenetics and genetically controlled development have been revolutionised during the last two decades. However, some of these evolutionary aspects are still debatable. MADS-box genes are known to play essential role in specifying the floral organogenesis and differentiation in numerous model plants like *Petunia hybrida*, *Arabidopsis thaliana* and *Antirrhinum majus*. SEPALLATA (SEP) genes, belonging to the MADS-box gene family, are members of the ABCDE and quartet models of floral organ development and play a vital role in flower development. However, few studies of the genes in *Prunus mume* have yet been conducted.

**Results:**

In this study, we cloned four *PmSEPs* and investigated their phylogenetic relationship with other species. Expression pattern analyses and yeast two-hybrid assays of these four genes indicated their involvement in the floral organogenesis with *PmSEP4* specifically related to specification of the prolificated flowers in *P. mume*. It was observed that the flower meristem was specified by *PmSEP1* and *PmSEP4*, the sepal by *PmSEP1* and *PmSEP4*, petals by *PmSEP2* and *PmSEP3*, stamens by *PmSEP2* and *PmSEP3* and pistils by *PmSEP2* and *PmSEP3*.

**Conclusion:**

With the above in mind, flower development in *P. mume* might be due to an expression of SEP genes. Our findings can provide a foundation for further investigations of the transcriptional factors governing flower development, their molecular mechanisms and genetic basis.

**Electronic supplementary material:**

The online version of this article (doi:10.1186/s12870-016-0954-6) contains supplementary material, which is available to authorized users.

## Background

Flower emergence is a vast step in the evolutionary history of plants [1], and its diversification overtime has largely altered the interaction patterns of the plant kingdom [2]. Furthermore, floral structures are controlled by a number of environmental and genetic factors. In recent years, consistent strides have been made to uncover the molecular basis behind flowering [3].

*Prunus mume* Sieb. et Zucc. (Rosaceae, Prunoideae), a traditional ornamental plant, has been cultivated in China for more than 3,000 years. During this long period of domestication and cultivation, the phenotypic characteristics of its flowers (such as single petal, double petal, multi-sepals, multi-pistils and prolificated flowers) have revolutionised. These variations have added more ornamental value to *P. mume* and are also useful when studying floral organ development. A series of flower development models are proposed for specimen plans [4, 5]. Genetic control of flower identity has been largely affected by the ABC model [6]. According to this model, three different gene classes signal floral organogenesis. The outermost sepals are specified by the A class (*AP1* and *AP2*), petals are controlled by the combination of A and B (*AP3* and *P1*) and C class genes (*AG*) and the carpels are specified by C class genes [7, 8]. MADS-box genes are of vital importance for ascertaining the genetic basis of plant development [9]. Among these, E class genes play a significant role in flower development. Scientists have already carried out investigations of the MADS-box gene family and the cloning of C class genes in *P. mume* [10]; however, the molecular mechanisms behind flower organ development and morphology remain unclear. Therefore, an expression and functional analysis of SEP genes is required to uncover these processes. Transcriptional regulators encoded by MADS-box genes have critical role in flower organ development [11]. A series of genes controlling flower development in ornamental plants have been identified as a result of continuous research on MADS-box genes. In peaches (*Prunus persica*), five MADS-box genes (*PpMADS1*, *PpMADS10*, *PrpMADS2*, *PrpMADS5* and *PrpMADS7*) have been cloned [12, 13]. Among these, *PrpMADS2*, *PrpMADS5* and *PrpMADS7* are homologous to SEP genes and have been shown to be preferentially expressed in flowers and fruit and to have the expression features of E class genes. Furthermore, the overexpression of these SEP genes in *Arabidopsis* produces different phenotypes. However, there is no phenotypic difference between the *PrpMADS2*-transgenic type and wild type in *Arabidopsis*; the overexpression of *PrpMADS5* and *PrpMADS7* can cause early blossoming. In addition, the early blossoming phenotype of *PrpMADS2*-transgenic plants is more powerful, and its extreme phenotype shows blooming even after germination [12]. Two C class genes (*CeMADS1* and *CeMADS2*) of *Cymbidium ensifolium* have been cloned and shaped into dimers after mixing with E class genes using yeast two-hybrid tests [14]. In another orchid, *Phalaenopsis*, four E class genes, belonging to the PeSEP1/3 and PeSEP2/4 branch, are expressed in all floral organs. In addition, these can form heterodimers with B, C, D and AGL6 proteins. Sepals of *Phalaenopsis* turn leafy when *PeSEP3* is silent, but there is no function in the flower phenotype when PeSEP2 is silent [15]. In *Arabidopsis thaliana*, four E class genes are indispensable in determining the flower organs and meristem [16–19]. Similarly, there are four E class genes (*PmMADS28*, *PmMADS17*, *PmMADS14* and *PmMADS32*) in the *P. mume* [10].

In the present study, we first identified and cloned four *PmSEPs* and then ascertained the functions of these genes in flower development to formulate a model for describing the genetic basis of floral organ development in *P. mume*. This study will set the foundation for a deep analysis of MADS-box genes in flower development and will provide a practical and effective way to improve the ornamental characteristics of *P. mume* using molecular methods.

## Methods

### Plant material

Three cultivars of *P. mume* with different flower types, ‘Jiang Mei’, ‘Sanlun Yudie’ and ‘Subai Taige’ (Additional file 1: Figure S1), were selected from the Jiufeng International Plum Blossom Garden, in Beijing, China (40° 07′ N, 116° 11′ E). Flower buds at different development stages (S1–S9) were harvested from each cultivar. After every 5–7d, samples of basic consistent appearance were collected. One of the samples was used to define the stages of flower bud development via paraffin sectioning, and the remaining samples were used for RNA extraction. Ten samples of different organs (root, stem and leaf during vegetative growth; sepal, petal, stamen and pistil of flower buds; and Fr1, Fr2 and Fr3 stages of fruit development corresponding to 10, 45 and 90 days after blooming, respectively) were taken from ‘Sanlun Yudie’. The pistils of ‘Jiang Mei’ and ‘Sanlun Yudie’, along with the variant pistil of ‘Subai Taige’, were sampled from the fourth floral whorl. All samples were quickly frozen in liquid nitrogen and stored at −80 °C until RNA extraction.

### Identification and cloning of SEP genes

Four *PmSEPs* were identified in our previous study [10]. On the basis of CDS sequences annotated in the genome database, PrimerPremier 5.0 was used to design specific primers. Total RNA was isolated from flower buds of ‘Sanlun Yudie’ using TRIzol reagent (Invitrogen, USA) following the manufacturer’s instructions. To remove potentially contaminating genomic DNA, RNA was treated with RNase-free DNase (Promega, USA). First-strand complementary DNA (cDNA) was synthesised from 2 μg total RNA with the TIANScript First Strand cDNA Synthesis Kit (Tiangen, China) following the manufacturer’s protocols. Full-length cDNA was obtained by performing PCR reactions in a 50 μl volume including 2 μl of cDNA, 10 μM of each primer (Additional file 2: Table S1), 0.4 μl Taq enzyme (Promega, USA) and 10 μl of PCR buffer. The thermal parameters were set to the following limits: 5 min at 94 °C; 30 cycles of 30 s at 94 °C, 30 s at annealing temperature (Additional file 2: Table S1), 1 min at 72 °C; ending 7 min at 72 °C and preservation at 4 °C. All target fragments were recovered by Gel Extraction Kit (Biomiga, USA) and were cloned into the pMDTM18-T vector (TaKaRa, China) to transform DH5α (Tiangen, China). PCR-positive colonies were sequenced by Taihe Biotechnology Co., Ltd.(China). The plasmids were extracted by Plasmid Miniprep Kit I (Biomiga, USA) and were stored at −80 °C. The cDNA sequences of four *PmSEP*s are shown in Additional file 3 (Data S1). The plasmids of three B class genes and one C class gene were obtained from previous experiments.

### Phylogenetic analyses

The Clustal X 2.0 program was used to perform multiple protein sequence alignment of four *PmSEPs* and 23 E-type genes in other plants (two *P. persica* genes, four *Malus domestica* MADs-box genes, two *Vitis vinifera* MADs-box genes, three *Actinidia chinensis* SEP genes, one *Lotus japonica* SEP gene, two *Oryza sativa* MADs-box genes, two *Petunia hybrida* FBP genes, four *A. thaliana* SEP genes, one *Zea mays* MADs-box gene and one *Fragaria ananassa* MADs-box gene) [20]. To study the phylogenetic relationships of SEP genes, several genes (four *P. mume* SEP genes, six *M. domestica* SEP genes, five *P. hybrida* FBP genes, four *A. thaliana* SEP genes and 23 E-type genes in other plants) were used to generate a phylogenetic tree using MEGA7.1 software with the maximum-likelihood (ML) method. The bootstrap values were set for 1,000 replicates, and the other parameters were set to default.

### Real-time quantitative RT-PCR

To analyse the expression profiles of SEP genes in flower buds at different development stages and in different organs, real-time RT-PCR experiments were performed using the PikoReal real-time PCR system (Thermo Fisher Scientific, Germany). A mix of 10 μl was made consisting of 2 μl cDNA, 2 μM of each primer (Additional file 4: Table S2) and 5 μl SYBR Premix ExTaq II (Takara, China). Temperatures were set as follows: 95 °C for 30 s; 40 cycles of 95 °C for 5 s, 60 °C for 30 s, 60 °C for 30 s; ending 20 °C. Furthermore, the temperature of the melting curve in these reactions was set to 60 °C ~ 95 °C, rising by 0.2 °C/s. Three biological duplications were performed in all real-time RT-PCR experiments, and each duplication was measured in triplicate. In these experiments, the reference gene was the protein phosphatase 2A (*PP2A*) and the relative expression levels were calculated using the2 ^– ΔΔCt^ method [21].

### Yeast two-hybrid assays

Full-length cDNA of all *PmSEPs* were amplified with gene-specific primers (Additional file 5: Table S3) via the PCR method. These amplified sequences were cloned into the pGBKT7 bait vector (Clonetech, USA) and pGADT7 prey vector (Clonetech, USA) using an In-Fusion HD Cloning Kit System at the EcoRI and BamHI sites. Subsequently, the bait vectors were transformed into yeast strain Y2H gold (Clonetech, USA), and the prey vectors into yeast strain Y187 (Clonetech, USA) using the Yeastmaker Yeast Transformation System 2 (Clonetech, USA). Later, these were selected on SD plates deficient of Trp and Leu. After that, single colonies of each transformant in checked SD medium were cultured overnight (30 °C, 250 rpm). Bait clones were tested for their autoactivation and toxicity. For subsequent interactions, two selective strains were mated with each other in YPDA liquid medium at 30 °C and 80 rpm for 20–24 h. The diploid mating bacterial liquid, which had been observed to have a cloverleaf structure using a 40 × microscope, was cultured on DDO plates (SD/-Trp/-Leu) at 30 °C for 3–5 d. Single colonies were chosen for culturing in DDO liquid medium. After growing at 30 °C, 250 rpm for 20–24 h, 700 g of bacterial liquid was centrifuged for 2 min, and the supernatant liquid was discarded. Next, 1.5 ml aseptic ddH2O was added to suspend sedimentary bacteria, and the previous operation was repeated. Afterward, sufficient aseptic ddH2O was added to make the OD_600_ of the bacterial liquid equal to 0.8. Finally, 100 μl of bacterial liquid (1, 1/10, 1/100 and 1/1000) was cultured on several DDO and QDO/X/A plates (SD/-Leu/-Trp/-His/-Ade/X-α-Gal/Aba) at 30 °C for 3–5 d. The screenings for protein-protein interaction events were implemented in triplicate.

## Results

### Identification and cloning of SEP genes in *P. mume*

There are four E class genes in the *P. mume* genome. According to their positions in the phylogenetic tree of SEP genes, they are *PmSEP1*, *PmSEP 2*, *PmSEP 3* and *PmSEP 4.* In order to obtain the sequences of the SEP genes, RT-PCR experiments were carried out to clone these genes. The CDS sequences of *PmSEP1*, *PmSEP2*, *PmSEP3* and *PmSEP4* were of 756 bp, 741 bp, 723 bp and 750 bp, encoding 251, 246, 240 and 249 amino acids, respectively. Based on the BLAST analysis, these sequences showed high similarity and consistency to their orthologues in other species. Additionally, all *PmSEP*s contained conserved MADS and K domains, belonging to the representative type IIMADS-box genes. Therefore, all results suggest that these four genes are E class genes.

### Multiple sequence alignment and phylogenetic analyses

**Fig. 1:**
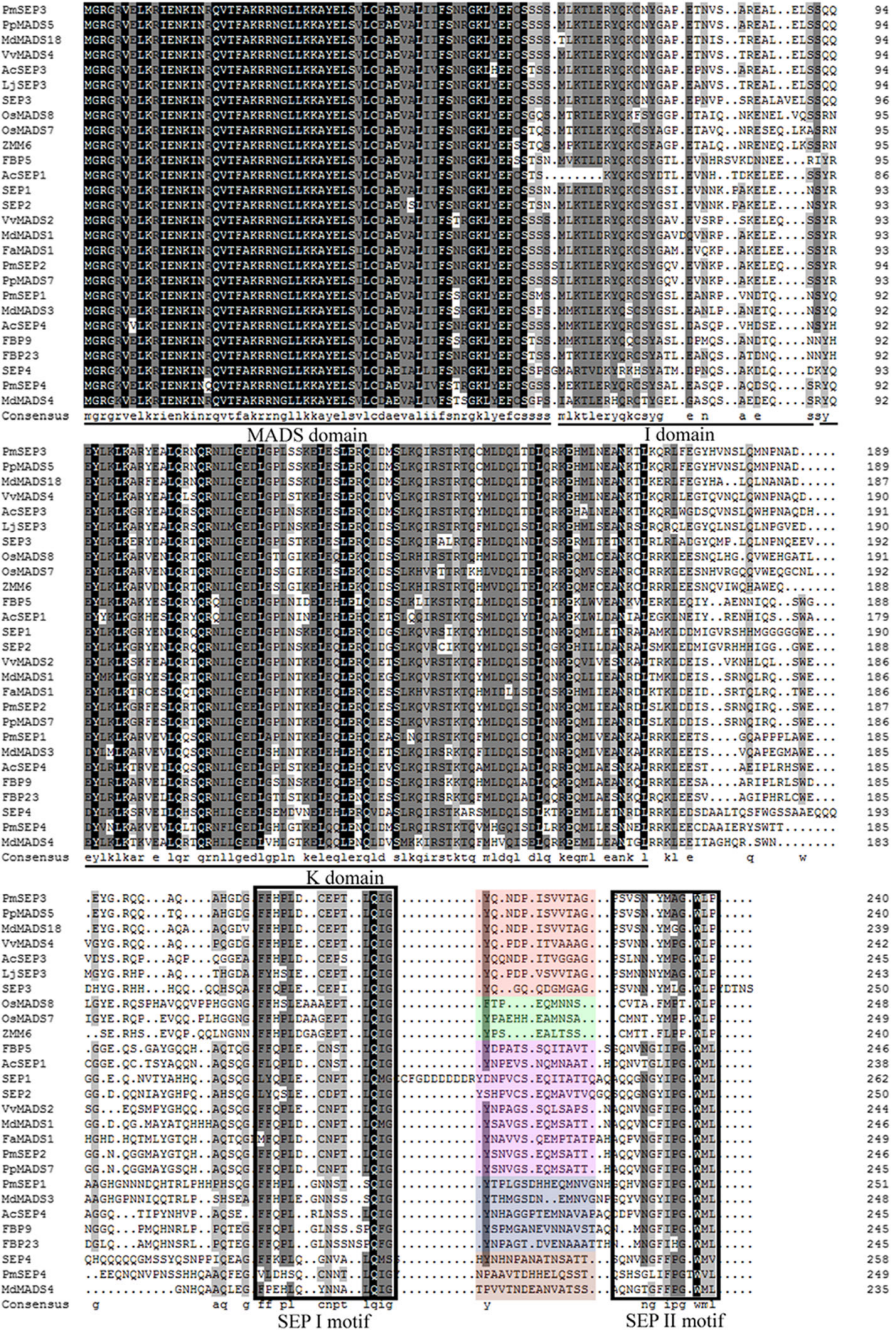
Multiple sequences alignment of E-class genes from *P. mume* and other species. The MADS, I and K domains are shown by lines on bottom of the alignment; two motifs of SEP genes are boxed; color shade box indicates lineage-specific motifs. The Gene Bank accession numbers of genes used in alignment are shown in Additional file 7 (Data S2)

The results of the multiple sequence alignment of the E class genes are shown in Fig. 1. In *PmSEPs*, the MADS domain was highly conservative, while the K domain was moderately conservative and the I domain showed little tendency toward conservatism. Consistent with previous studies, there were two conserved motifs, SEP I and SEP II, in the C-terminal. In addition, a conserved motif of a specific evolutionary branch between these two SEP motifs was also found. The C-terminal of SEP genes exhibited low conservancy among different evolutionary branches, but these fragments were highly conservative in the same branch.

**Fig. 2:**
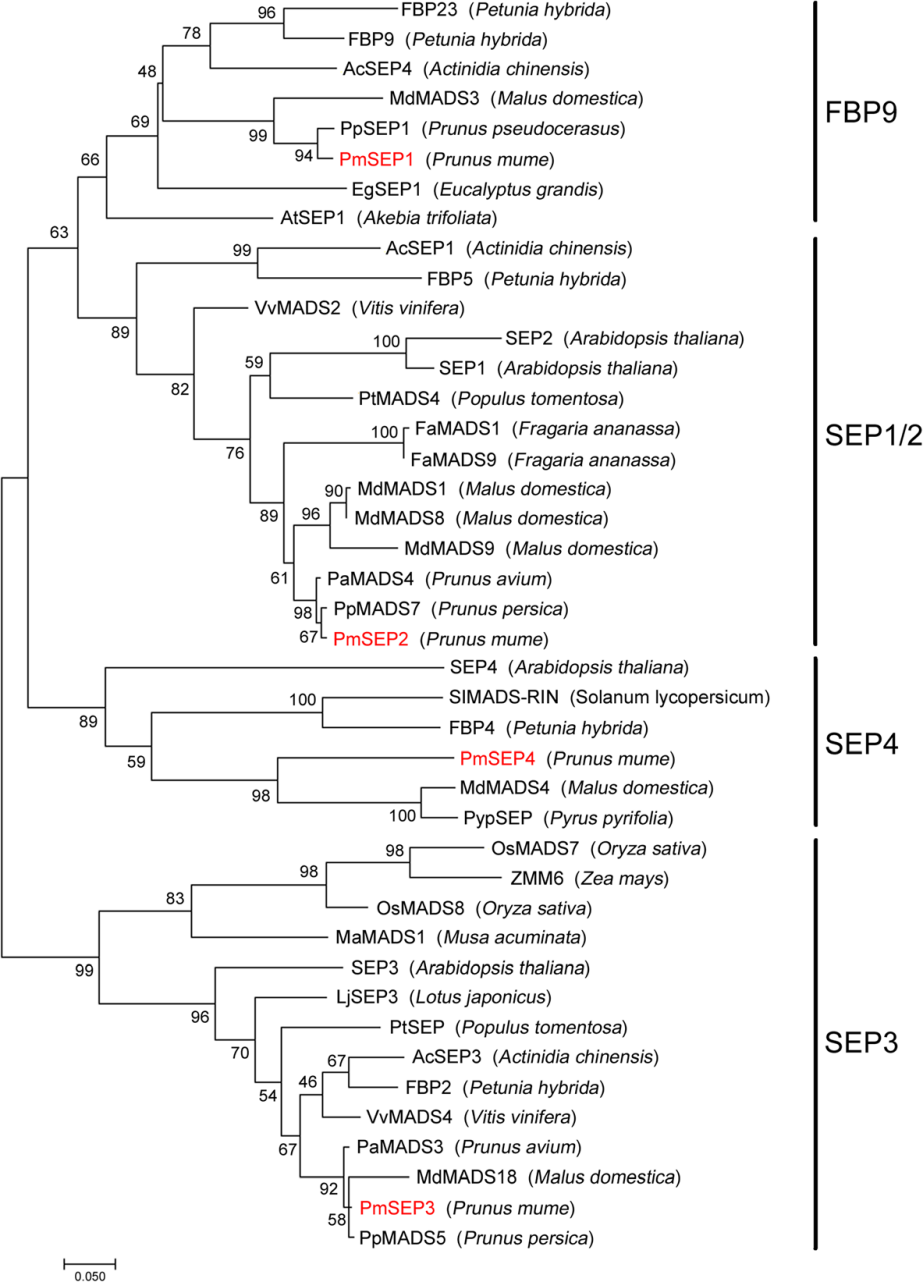
Phylogenetic tree of E-class MADS-box proteins from *P. mume* and other species. The Gene Bank accession numbers of genes used in constructing phylogenetic tree are shown in Additional file 8 (Data S3)

According to the phylogenetic tree (Fig. 2) of SEP genes, four evolutionary branches (SEP3, SEP1/2, FBP9 and SEP4 clades) were identified. Four E class genes of *P. mume* were clustered with SEP genes from other *Prunus* or Rosaceae plants. These results suggest that these four *PmSEPs* evolved from primitive Rosaceae plants, rather than from their own duplicative events.

### Expression analyses

In order to ascertain the role of SEP genes in organogenesis and floral organ development, the expression patterns of the *PmSEP*s in different organs (root, stem, leaf, four whorls of flower buds and three stages of fruits) and nine stages of flower development were studied using quantitative RT-PCR.

**Fig. 3:**
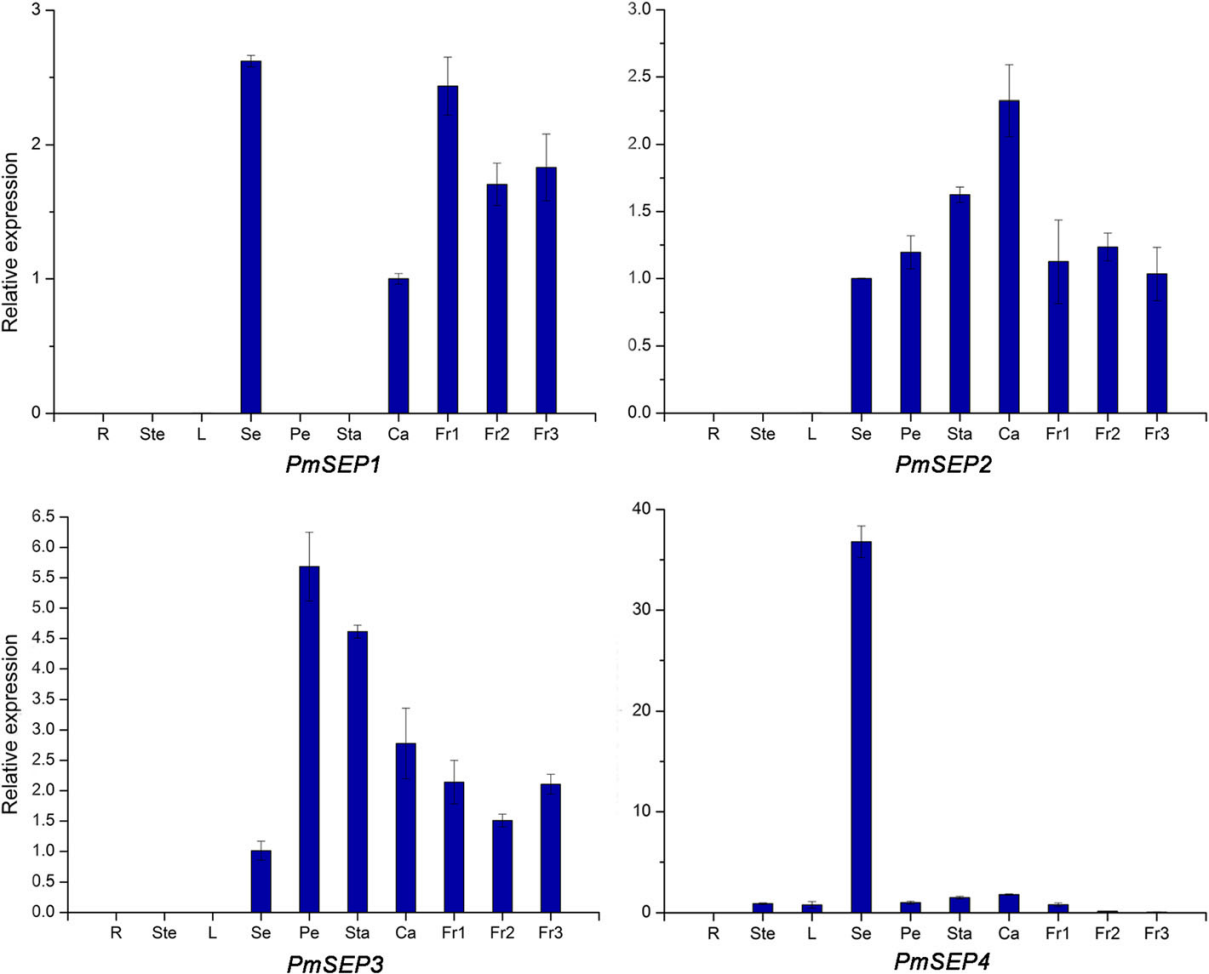
Expression patterns of the E-class MADS-box genes in different organs of *P. mume*. R: Root, Ste: Stem, L: Leaf, Se: Sepal, Pe: Petal, Sta: Stamen, Ca: Carpel, Fr1-3: Fruit development stages 1–3

These four *PmSEP*s exhibited various expression profiles. They were highly expressed in flowers and fruits (Fig. 3). The expressions of *PmSEP2* and *PmSEP3* were restricted to flowers and fruits, but the transcripts of *PmSEP1* and *PmSEP4* were mildly detected in vegetative organs. Furthermore, both *PmSEP2* and *PmSEP3* were expressed in all floral organs, with predominantly high expression levels being observed in the pistil and petal, respectively. Compared with this, *PmSEP1* was expressed only in the sepal and pistil, and the expression of *PmSEP4* was notably detected in the sepal and showed faint expression in fruit and other organs. *PmSEP1*, *PmSEP2* and *PmSEP3* were all highly expressed in the fruit stages. In addition, *PmSEP1* and *PmSEP3* were down-regulated in the Fr2 stage and up-regulated in the Fr3 stage, while *PmSEP2* was up-regulated in the Fr2 stage and down-regulated in the Fr3 stage.

**Fig. 4:**
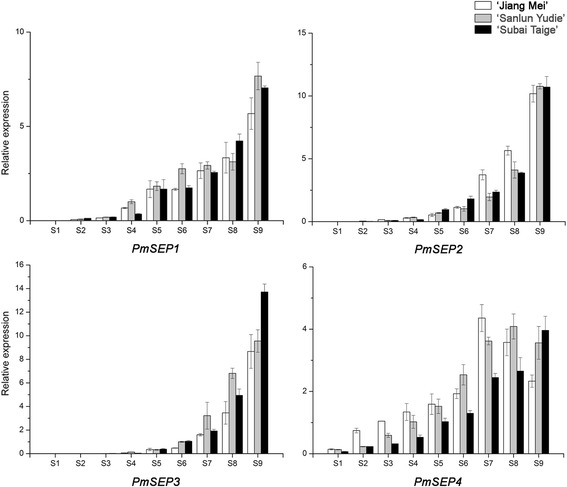
Expression patterns of E class MADS-box genes during *P. mume* floral bud differentiation

Based on the paraffin section analyses (Additional file 6: Figure S2), there were nine development stages (S1–S9) of flower buds in *P. mume*, including: undifferentiation (S1), flower primordium formation (S2), sepal initiation (S3), petal initiation (S4), stamen initiation (S5), pistil initiation (S6), stamen and pistil elongation (S7), ovule development (S8) and anther development (S9). All *PmSEPs* demonstrated different expression profiles in flower development (Fig. 4). Their expression levels continuously increased during flower bud differentiation and were the highest in S9. *PmSEP4* was expressed in all nine stages, while *PmSEP1–3* had stage-specific expression behaviours. Transcription of *PmSEP1* was expressed during S2 through S9, which shows its association with the specification of flower primordium. *PmSEP2* and *PmSEP3* began to express during S3 and S4, respectively, suggesting their participation in the development of specific floral organs. In different cultivars, the expression levels of *PmSEP1* and *PmSEP2* showed little variation. *PmSEP3* had similar expression profiles during S4–S8, but its impression was higher in ‘Subai Taige’ as compared with ‘Jiang Mei’ and ‘Sanlun Yudie’ in S9. *PmSEP4* was up-regulated during S1–S7 and down-regulated during S7–S9 in ‘Jiang Mei’. Similarly it was up-regulated during S1–S8 and down-regulated during S8–S9 in ‘Sanlun Yudie’ and unceasingly up-regulated during S1–S9 in ‘Subai Taige’. Additionally, during S1–S8, the expression levels of *PmSEP4* were comparatively higher in ‘Jiang Mei’ and ‘Sanlun Yudie’ than in ‘Subai Taige’. Nevertheless, in S9, *PmSEP4* was more prominent in ‘Subai Taige’ as compared with ‘Jiang Mei’ and ‘Sanlun Yudie’.

**Fig. 5:**
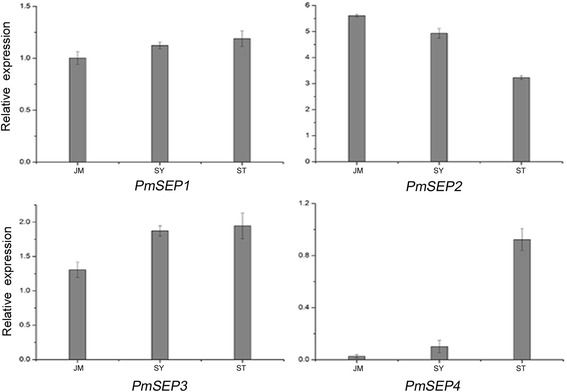
Expression patterns of E class MADS-box genes in the fourth whorl of different flower types of *P. mume*. JM: ‘Jiang Mei’; SY: ‘Sanlun Yudie’; ST: ‘Subai Taige’

SEP genes were divided into two groups according to their expression patterns in the fourth floral whorl tissues of different flower types. One group contained three genes (*PmSEP1*, *PmSEP2* and *PmSEP3)* with similar expression profiles in different cultivars. The other group had only one gene, *PmSEP4*, which was prominent in ‘Subai Taige’ but poorly expressed in ‘Jiang Mei’ and ‘Sanlun Yudie’ (Fig. 5), indicating that it might be concerned with the formation of upper flower in duplicated flowers.

### Protein-protein interactions among SEP genes in *P. mume*

**Fig. 6:**
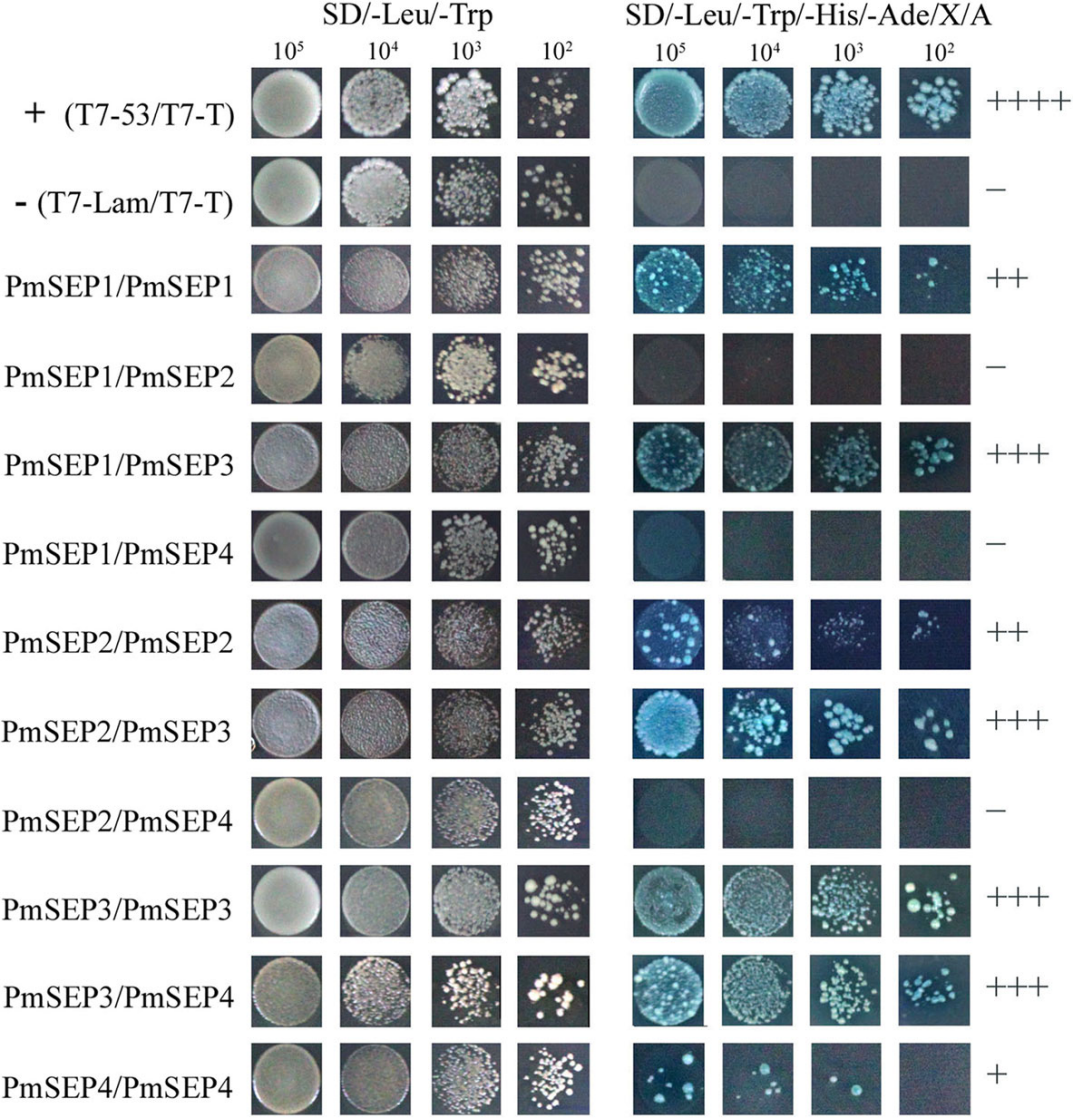
Protein-protein interactions between *P. mume* E class MADS-box genes

We performed yeast two-hybrid assays of four SEP genes, three B class genes and one C class gene in *P. mume*, to investigate the protein-protein interaction relationships among genes. Although *P. mume* and *A. thaliana* had four SEP members, their evolutionary processes were quite different. Thus, the interaction model of the four *PmSEP*s might be quite different from their orthologues in *A. thaliana*. The results of dimerisation among four *PmSEP*s are shown in Fig. 6. *PmSEP1*, *PmSEP2* and *PmSEP4* could interact with each other, and all of them could interact with *PmSEP3*. These results suggest that all *PmSEP*s can form both homodimers and heterodimers with *PmSEP3*. These three heterodimers showed strong, yet unequal interactive capability; *PmSEP1*, *PmSEP2* and *PmSEP3* showed stronger interactive capability to form homodimers than *PmSEP4*.

**Fig. 7:**
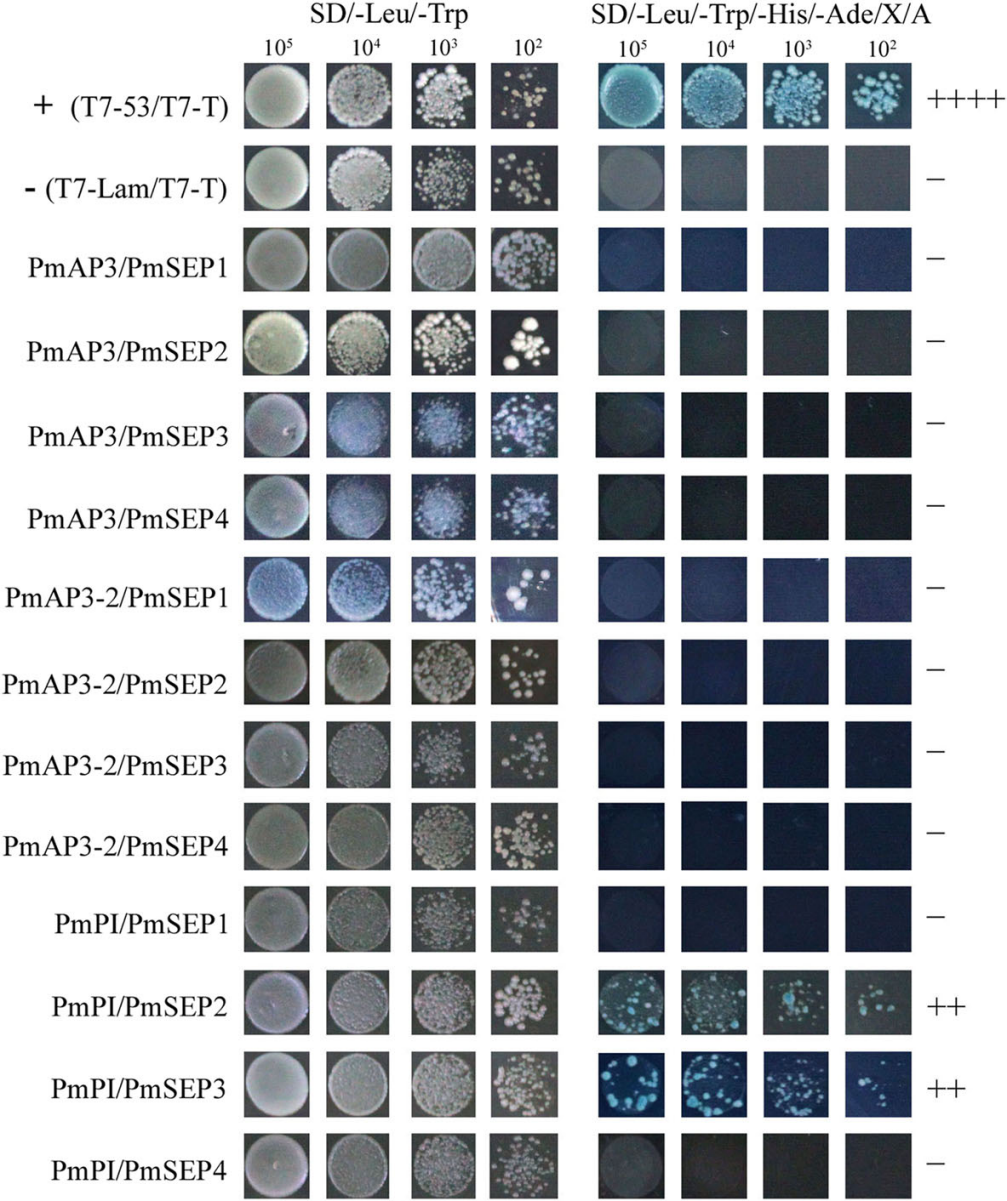
Protein-protein interactions between *P. mume* B class genes and E class MADS-box genes

**Fig. 8:**
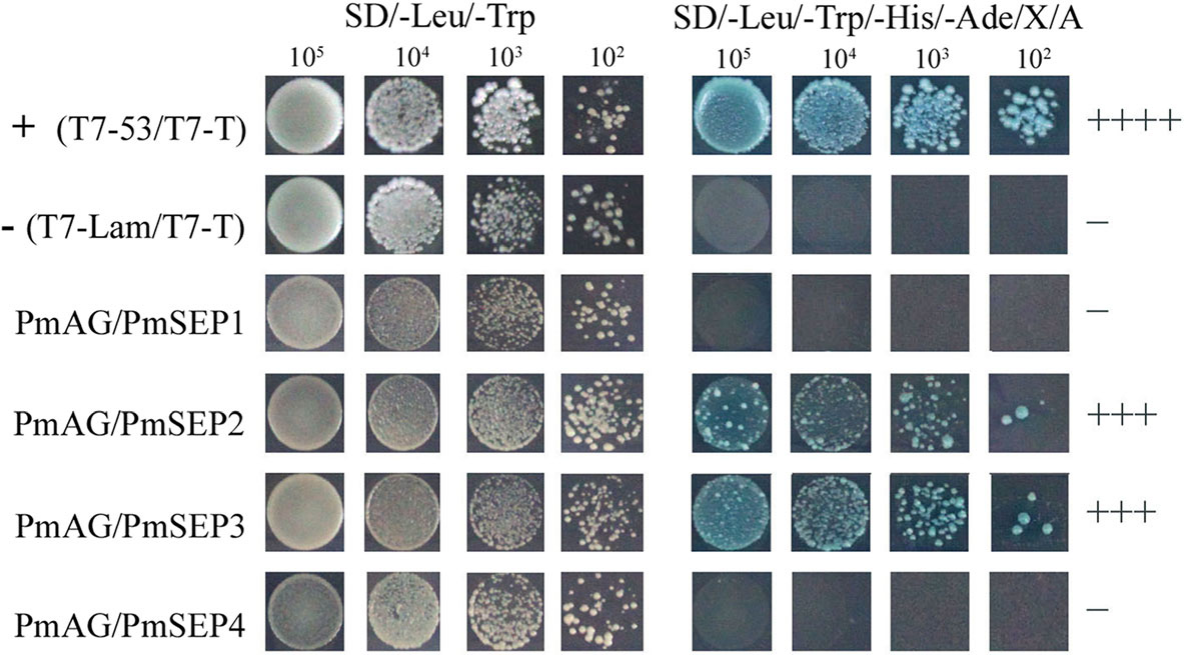
Protein-protein interactions between *P. mume* C class genes and E class MADS-box genes

There were few B class genes in *P. mume* that could interact with the four *PmSEP*s (Fig. 7). Only found one B class gene, *PmPI*, exhibited strong interaction with *PmSEP2* and *PmSEP3*. None of the two AP3-type genes could interact with any *PmSEP*s. The complexes formed by B class genes with SEP-like genes were combined by *PmPI*. Figure 8 shows the interaction patterns of the four E class genes with one C class gene in *P. mume*. Only two SEP genes, *PmSEP2* and *PmSEP3*, could strongly dimerise with *PmAG*. The dimerisation properties and expression analyses may help to identify SEP protein pairs that function together and may provide a basis for further investigation into these functional redundancies in the overlapping interaction maps.

## Discussion

MADS-box genes only exist in Eudicotyledons [22]. In *A. thaliana*, there are four E class genes (*AtSEP1*–*4*) that play pronounced roles in the flower meristem and flower organs determinacy with redundant function [16–19, 23]. Similarly, we found four SEP genes (*PmSEP1–4*) in *P. mume*. The SEP genes of plants are clustered into four evolutionary branches: SEP3 clade, SEP1/2 clade, FBP clade and SEP4 clade. Previous studies have suggested that E class MADS-box genes are involved in floral organ development, and their expression patterns vary [23]. In *A. thaliana*, *AtSEP1* and *AtSEP2*, both of which belong to the SEP1/2 clade, are duplicate genes; *AtSEP3* is in the SEP3 clade. The transcripts of *AtSEP1*, *AtSEP2* and *AtSEP3* were only detected in floral organs and were restricted to the second, third and fourth floral whorl; *AtSEP4* was expressed in the fourth floral whorl and the vegetative organs [16, 24–26]. In *P. mume*, *PmSEP2* was in the SEP1/2 clade and *PmSEP3* was in the SEP3 clade. The transcripts of *PmSEP2* and *PmSEP3*, similar to their homologues in *A. thaliana*, were not detected in vegetative organs. However, these genes were expressed not only in floral organs but also in fruit, indicating that they may function differently with their homologues in *A. thaliana*. The same phenomenon was also found in strawberries (*Fragaria* x *ananassa* Duch.), apples (*Malus* x *domestica*) and poplars (*Populus tremuloides*). *FaMADS9*, a member of the SEP1/2 clade in strawberries, is expressed in petals, the thalamus and fruit [27]. In apples, two genes of the SEP1/2 clade, *MdMADS8* and *MdMADS9*, are expressed in both flowers and fruit [28]. The transcript of PTM3/4, belonging to the SEP1/2 clade in poplars, is detected in buds, leaves, stems and flowers; however, in the SEP3 clade, *PTM6* is only expressed in flowers [29]. Conversely, the SEP4 clade gene in *A. thaliana*, *AtSEP4*, is the only gene expressed in the flower, fruit and vegetative organs simultaneously. *SlMADS-RIN*, the homologous gene of *AtSEP4*, is necessary for fruit ripening in tomatoes (*Solanum lycopersicum*) [30]. *MdMADS4*, a member of the SEP4 clade in apples, is expressed in four floral whorls and fruit [31]. In *P. mume*, the transcript of *PmSEP4* was detected in all organs, but only showed high expression level in sepals, which is indicative of its participation in sepal development. In the case of strawberries, the expression level of *FaMADS4* is low during fruit development [27]. The general conclusion is that the expression patterns of SEP genes in the same clade can show both conservation and divergence, depending on the species within which they are being observed.

*PmSEP1* was clustered in the FBP9 clade, which is not present in *A. thaliana* [32]. In addition, the expression level of *PmSEP1* was high in sepals, pistil and fruit, but was low in vegetative organs. In line with our findings, *PrpMADS2*, the homologue of *PmSEP1*, is expressed in sepals, pistils, fruits and petals [12]. The expression profiles of SEP genes in the same clade were different in the different species, which is indicative of their evolutionary functional divergence [22]. This is due to the fact that multiple SEP genes exist in the plant genome (e.g., the expression level of *PmSEP4* was low in fruits, but *PmSEP1*, *PmSEP2* and *PmSEP3* were highly expressed). The *SEP3* orthologue holds a major role in the development of pistil in Ranunculates [23]. All of these *PmSEP*s were expressed prominently in reproductive parts, justifying their key role in flower and fruit development.

Prolificated flowers are a very special flower type in *P. mume* wherein the fourth whorl of floral organ, which should be pistils, is differentiated into sepals or even a complete upper flower. According to the expression patterns of the four *PmSEP*s, we found that only *PmSEP4* was more highly expressed in the fourth floral whorl of ‘Subai Taige’ than in the other two cultivars, which had no prolificated flowers. Furthermore, the expression level of *PmSEP4* was notably high in sepals, but low in other organs; we can, therefore, speculate that *PmSEP4* is somehow linked with the formation of the upper flower in *P. mume*. Based on the expression patterns of SEP genes, it can be concluded that *PmSEP2*, *PmSEP3* and *PmSEP4* are involved in the development of all four floral whorls, while *PmSEP1* only specifies sepals and pistils. In addition, *PmSEP1* and *PmSEP4* might affect the flower’s primordium formation. The expression profiles of the four *PmSEP*s in flower bud differentiation were consistent with their specific expression patterns corresponding with floral organs, and their expression profiles in different cultivars were similar.

In the analyses of the protein-protein interactions among eight MADS-box genes, four E class genes could form dimers with other genes and act as ‘glue’ to make combinations with other dimers, thereby forming a polymer [15, 33]. According to the ‘floral quartet models’ of floral organ development, B, C, and E class proteins act together to determine the characteristics of stamens while the tetramer of two C class proteins and two E class proteins determine the characteristics of the pistil. Previous studies have shown that *AtSEP3* plays an essential role in DNA bending, thus forming cyclic tetramers [34]. In *P. mume*, *PmSEP2* and *PmSEP3* could form dimers with B and C class genes, showing that these two SEP genes might participate in petal, stamen and pistil development. However, *PmSEP1* and *PmSEP4* could not form any heterodimers with B and C class genes. Moreover, due to their high expression level in sepals, it is likely that *PmSEP1* and *PmSEP4* are concerned with sepal development. According to studies in the expression patterns, protein-protein interaction profiles and comparative analyses of SEP genes with their orthologues, the roles of SEP genes in controlling floral organ development in *P. mume* have been proposed. We can now suggest the molecular regulation model of SEP genes in floral organ development in *P. mume*: *PmSEP1* and *PmSEP4* specify the flower meristem and sepal; petals are controlled by *PmSEP2* and *PmSEP3*; stamens are specified by *PmSEP2* and *PmSEP3* and carpel is controlled by *PmSEP2* and *PmSEP3*. Furthermore, for prolificated flowers, it is possible that *PmSEP4* is involved in the formation of the upper flower in *P. mume*.

In this study, we first cloned four SEP genes in *P. mume* and then investigated their expression patterns and protein-protein interactions. All results were used to elucidate the roles of these genes in *P. mume* flower development and proposed a molecular regulation model for flower organ development. This work sets the foundation for further research on the functions of SEP genes during flower organ development. In the future, we will transfer these four genes into *A. thaliana* to verify their function, which will improve the molecular model of floral organ development.

## Conclusion

Despite its immense importance, functional studies pertaining to the genetic control of flower characterisation are rare in *P. mume.* The comprehensive exploration of floral SEP genes can do a great deal to expand the understanding of the genetic basis behind flower development and its prolification in *P. mume*. To the best of our knowledge, this is a novel investigation ascertaining the role of SEP genes in floral expression and the floral organogenesis of *Prunus*. Our research gives insight into the development of prolificated flowers, thus broadening the genetic basis of flower evolution.

## Additional files

Additional file 1: Figure S1. Flower of P. mume. From figure 1 to figure 3 successively were ‘Jiang Mei’, ‘Sanlun Yudie’ and ‘Subai Taige’. (DOCX 69 kb)
Additional file 2: Table S1. Primers used for cloning. (DOCX 14 kb)
Additional file 3: Data S1. The sequences of four Prunus mume SEP genes. (DOCX 14 kb)
Additional file 4: Table S2. Primers used for real-time quantitative RT-PCR. (DOCX 14 kb)
Additional file 5: Table S3. Primers used in PCR reaction. (DOCX 14 kb)
Additional file 6: Figure S2. Flower bud differentiation of P. mume. The flower bud development was divided into eight stages (S1-9): undifferentiation (S1), flower primordium formation (S2), sepal initiation (S3), petal initiation (S4), stamen initiation (S5), pistil initiation (S6), stamen and pistil elongation (S7), ovule development (S8), anther development (S9). The letters had different meanings. FP: Flower primordium; SeP: Sepal primordium; Se: Sepal; PeP: Petal primordium; Pe: Petal; StP: Stamen primordium; St: Stamen; CaP: Carpel primordium; Ca: Carpel; Sty: Style; An: Anther; F: Filament; Ova: Ovary; Ovu: Ovule; Po: Pollen. (DOCX 217 kb)
Additional file 7: Data S2. The GeneBank accession numbers of genes used in alignment. (DOCX 13 kb)
Additional file 8: Data S3. The GeneBank accession numbers of genes used in constructing phylogenetic tree. (DOCX 13 kb)

## Abbreviations

cDNA: Complementary DNA
ML: Maximum likelihood
PmSEPs: *Prunus mume SEP* genes
*PP2A*: protein phosphatase 2A
*SEP*: *SEPALLATA*
Y2H: Yeast two-hybrid

## References

[1] Magallón S, Gómez-Acevedo S, Sánchez-Reyes LL, Hernández-Hernández T (2015). A metacalibrated time-tree documents the early rise of flowering plant phylogenetic diversity. New Phytol.

[2] Chanderbali AS, Berger BA, Howarth DG, Soltis PS, Soltis DE (2016). Evolving Ideas on the Origin and Evolution of Flowers. New Perspectives in the Genomic Era. Genetics.

[3] Oh M, Lee U (2007). Historical perspective on breakthroughs in flowering field. J Plant Biol.

[4] Causier B, Schwarz-Sommer Z, Davies B (2010). Floral organ identity: 20 years of ABCs. Semin Cell Dev Biol.

[5] Theissen G (2001). Development of floral organ identity. stories from the MADS house. Curr Opin Plant Biol.

[6] Acri-Nunes-Miranda R, Mondragón-Palomino M (2013). Expression of paralogous *SEP*-, *FUL*-, *AG*- and *STK*-like MADS-box genes in wild-type and peloric *Phalaenopsis* flowers. Front Plant Sci.

[7] Yoon HS (2003). A floral meristem identify gene influences physiological and ecological aspect of floral organogenesis. J Plant Biol.

[8] Li Q, Huo Q, Wang J, Jing Z, Sun K, He C (2016). Expression of B-class MADS-box genes in response to variations in photoperiod is associated with chasmogamous and cleistogamous flower development in *Viola philippica*. BMC Plant Biol.

[9] Kim SH, Hamada T, Otani M, Shimada T (2005). Isolation and characterization of MADS box genes possibly related to root development in sweetpotato (*Ipomoea batatas* L. Lam.). J Plant Biol.

[10] Xu Z, Zhang Q, Sun L, Du D, Cheng T, Pan H, Yang W, Wang J (2014). Genome-wide identification, characterisation and expression analysis of the MADS-box gene family in *Prunus mume*. Mol Genet Genomics.

[11] Tani E, Polidoros AN, Flemetakis E, Stedel C, Kalloniati C, Demetriou K, Katinakis P, Tsaftaris AS (2009). Characterization and expression analysis of *AGAMOUS* -like, *SEEDSTICK* -like, and *SEPALLATA* -like MADS-box genes in peach ( *Prunus persica* ) fruit. Plant Physiol Biochem.

[12] Xu Y, Zhang L, Xie H, Zhang Y-Q, Oliveira MM, Ma R-C (2008). Expression analysis and genetic mapping of three *SEPALLATA*-like genes from peach (*Prunus persica* (L.) Batsch). Tree Genet Genomes.

[13] Zhang L, Xu Y, Ma R (2008). Molecular cloning, identification, and chromosomal localization of two MADS box genes in peach (*Prunus persica*). J Genet Genomics.

[14] Wang SY, Lee PF, Lee YI, Hsiao YY, Chen YY, Pan ZJ, Liu ZJ, Tsai WC (2011). Duplicated C-class MADS-box genes reveal distinct roles in gynostemium development in *Cymbidium ensifolium* (Orchidaceae). Plant Cell Physiol.

[15] Pan ZJ, Chen YY, Du JS, Chen YY, Chung MC, Tsai WC, Wang CN, Chen HH (2014). Flower development of *Phalaenopsis* orchid involves functionally divergent *SEPALLATA*-like genes. New Phytol.

[16] Ditta G, Pinyopich A, Robles P, Pelaz S, Yanofsky MF (2004). The SEP4 gene of *Arabidopsis thaliana* functions in floral organ and meristem identity. Curr Biol.

[17] Honma T, Goto K (2001). Complexes of MADS-box proteins are sufficient to convert leaves into floral organs. Nature.

[18] Pelaz S, Ditta GS, Baumann E, Wisman E, Yanofsky MF (2000). B and C floral organ identity functions require *SEPALLATA* MADS-box genes. Nature.

[19] Mandel AM, Yanofsky FM (1998). The *Arabidopsis AGL9* MADS box gene is expressed in young flower primordia. Sex Plant Reprod.

[20] Larkin MA, Blackshields G, Brown NP, Chenna R, McGettigan PA, McWilliam H, Valentin F, Wallace IM, Wilm A, Lopez R (2007). Clustal W and Clustal X version 2.0.. Bioinformatics.

[21] Wang T, Hao R, Pan H, Cheng T, Zhang Q (2014). Selection of Suitable Reference Genes for Quantitative Real-time Polymerase Chain Reaction in *Prunus mume* during Flowering Stages and under Different Abiotic Stress Conditions. Amer Soc Hort Sci.

[22] Zahn LM, Kong H, Leebens-Mack JH, Kim S, Soltis PS, Landherr LL, Soltis DE, Depamphilis CW, Ma H (2005). The evolution of the *SEPALLATA* subfamily of MADS-box genes: a preangiosperm origin with multiple duplications throughout angiosperm history. Genetics.

[23] Soza VL, Snelson CD, Hazelton KDH, Stilio VSD. Partial redundancy and functional specialization of E-class SEPALLATA genes in an early-diverging eudicot. Developmental Biology. 2016;419(1):143–55. 10.1016/j.ydbio.2016.07.021 27502434

[24] Savidge B, Rounsley SD, Yanofsky MF (1995). Temporal relationship between the transcription of two *Arabidopsis* MADS box genes and the floral organ identity genes. Plant Cell.

[25] Ma H, Yanofsky MF, Meyerowitz EM (1991). *AGL1-AGL6*, an *Arabidopsis* gene family with similarity to floral homeotic and transcription factor genes. Genes Dev.

[26] Flanagan CA, Ma H (1994). Spatially and temporally regulated expression of the MADS-box gene *AGL2* in wild-type and mutant *Arabidopsis* flowers. Plant Mol Biol.

[27] Seymour GB, Ryder CD, Cevik V, Hammond JP, Popovich A, King GJ, Vrebalov J, Giovannoni JJ, Manning K (2011). A *SEPALLATA* gene is involved in the development and ripening of strawberry (*Fragaria x ananassa* Duch.) fruit, a non-climacteric tissue. J Exp Bot.

[28] Ireland HS, Yao J-L, Tomes S, Sutherland PW, Nieuwenhuizen N, Gunaseelan K, Winz RA, David KM, Schaffe RJ (2013). Apple *SEPALLATA1/2*-like genes control fruit flesh development and ripening. Plant J.

[29] Cseke LJ, Cseke SB, Ravinder N, Taylor LC, Shankar A, Sen B, Thakur R, Karnosky DF, Podila GK (2005). SEP-class genes in *Populus tremuloides* and their likely role in reproductive survival of poplar trees. Gene.

[30] Vrebalov J, Ruezinsky D, Padmanabhan V, White R, Medrano D, Drake R, Schuch W, Giovannoni J (2002). A MADS-box gene necessary for fruit ripening at the tomato ripening-inhibitor (rin) locus. Science (New York, NY).

[31] Sung SK, Yu GH, Nam J, Jeong DH, An G (2000). Developmentally regulated expression of two MADS-box genes, *MdMADS3* and *MdMADS4*, in the morphogenesis of flower buds and fruits in apple. Planta.

[32] Malcomber ST, Kellogg EA (2005). *SEPALLATA* gene diversification: brave new whorls. Trends Plant Sci.

[33] Melzer R, Theissen G (2009). Reconstitution of ‘floral quartets’ in vitro involving class B and class E floral homeotic proteins. Nucleic Acids Res.

[34] Melzer R, Verelst W, Theissen G (2009). The class E floral homeotic protein *SEPALLATA3* is sufficient to loop DNA in ‘floral quartet’-like complexes in vitro. Nucleic Acids Res.

