# Supplementary material for: SEP-class genes in Prunus mume and their likely role in floral organ development
Source: BMC Plant Biol. 2017 Jan 13;17:10. doi: 10.1186/s12870-016-0954-6 (PMC5234111; doi:10.1186/s12870-016-0954-6)

**Additional file1**

**SEP-class genes in** ***Prunus mume* and their likely role in floral organ development**

Yuzhen Zhou, Zongda Xu, Xue Yong, Sagheer Ahmad, Weiru Yang, Tangren Cheng, Jia Wang, Qixiang Zhang*

*Affiliation*: Beijing Key Laboratory of Ornamental Plants Germplasm Innovation & Molecular Breeding, National Engineering Research Center for Floriculture, Beijing Laboratory of Urban and Rural Ecological Environment, Key Laboratory of Genetics and Breeding in Forest Trees and Ornamental Plants of Ministry of Education, School of Landscape Architecture, Beijing Forestry University, Beijing, 100083, China.

******Corresponding author*

Qixiang Zhang: zqxbjfu@126.com;

**FigureS1.** Flower of *P. mume*. From figure1 to figure 3 successively were ‘Jiang Mei’, ‘Sanlun Yudie’ and ‘Subai Taige’.


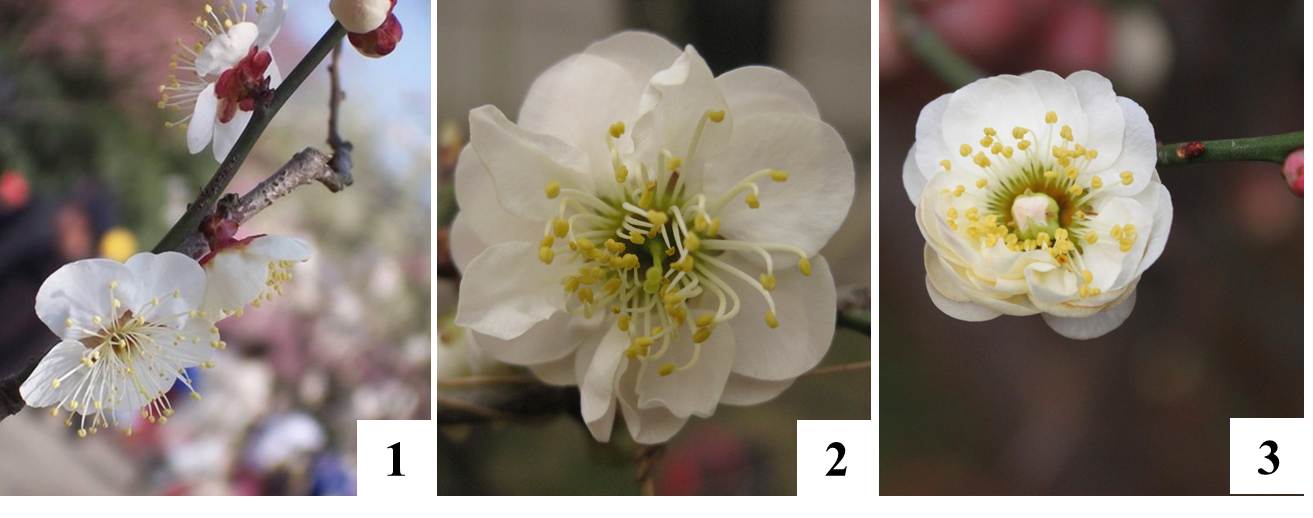

Supplement: Additional file 1: Figure S1. — Flower of P. mume. From figure 1 to figure 3 successively were ‘Jiang Mei’, ‘Sanlun Yudie’ and ‘Subai Taige’. (DOCX 69 kb) [file 12870_2016_954_MOESM1_ESM.docx]
