# Supplementary material for: SEP-class genes in Prunus mume and their likely role in floral organ development
Source: BMC Plant Biol. 2017 Jan 13;17:10. doi: 10.1186/s12870-016-0954-6 (PMC5234111; doi:10.1186/s12870-016-0954-6)
Supplement: Additional file 3: Data S1. — The sequences of four Prunus mume SEP genes. (DOCX 14 kb) [file 12870_2016_954_MOESM3_ESM.docx]

**Additional file3**

**SEP-class genes in** ***Prunus mume* and their likely role in floral organ development**

Yuzhen Zhou, Zongda Xu, Xue Yong, Sagheer Ahmad, Weiru Yang, Tangren Cheng, Jia Wang, Qixiang Zhang*

*Affiliation*: Beijing Key Laboratory of Ornamental Plants Germplasm Innovation & Molecular Breeding, National Engineering Research Center for Floriculture, Beijing Laboratory of Urban and Rural Ecological Environment, Key Laboratory of Genetics and Breeding in Forest Trees and Ornamental Plants of Ministry of Education, School of Landscape Architecture, Beijing Forestry University, Beijing, 100083, China.

******Corresponding author*

Qixiang Zhang: zqxbjfu@126.com;

**Data S1.** The sequences of four *Prunus mume* SEP genes.

>PmSEP1

ATGGGAAGAGGTAGAGTTGAGCTGAAGAGGATAGAGAATAAAATTAACAGGCAAGTGACTTTCGCCAAGAGAAGAAATGGGCTGCTCAAGAAAGCTTATGAGCTCTCAGTTCTCTGCGATGCTGAGGTTGCCCTCATTATTTTCTCCAGCCGTGGAAAACTTTATGAATTCTGTAGCAGTATGAGCATGCTGAAAACGCTTGAAAAGTACCAAAGGTGCAGCTATGGCTCCCTGGAAGCCAACAGACCCGTCAATGACACTCAGAACAGCTATCAGGAATATCTGAAGCTGAAAGCCAGAGTGGAGGTCCTCCAACAATCTCAAAGAAACCTTCTTGGGGAAGATTTGGCCCCACTGAACACAAAGGAGCTTGAGCAGCTTGAGCATCAACTGGAGGCATCCTTGAACCAAATTAGATCAACAAAGACTCAGTTTATGCTTGATCAGCTTTGTGATCTCCAGAACAAGGAACAAATGCTAGTTGAAGCTAACAAAGCCTTGAGGAGGAAGCTGGAAGAAACTAGTGGGCAAGCTCCACCTCCATTGGCATGGGAAGCTGCTGGCCATGGCAACAACAATGATCAGCATACTCGCCTTCCTCATCATCCTCACTCACAAGGCTTCTTCCATCCATTGGGAAACAACTCCACTTCCCAAATTGGATACACCCCTTTGGGTTCCGATCATCATGAACAAATGAATGTTGGAAATCATGGCCAACATGTGAATGGATTCATTCCTGGGTGGATGCTTTGA

>PmSEP2

ATGGGGAGGGGAAGAGTGGAACTCAAGAGGATAGAGAACAAGATAAACAGGCAAGTCACATTTGCAAAGAGAAGAAATGGGCTTCTGAAGAAAGCCTATGAGCTCTCTATTCTCTGCGATGCTGAGGTTGCTCTCATCATCTTCTCTAACCGTGGCAAGCTCTATGAGTTCTGTAGCAGCTCTAGTAGCATACTCAAAACACTGGAAAGGTACCAGAAGTGCAGTTATGGTCAAGTGGAAGTCAACAAACCTGCCAAGGAACTTGAGCAGAGCAGCTACCGCGAGTACTTGAAACTGAAAGGTCGATTTGAGTCCCTACAACGAACTCAGAGAAACCTTCTTGGAGAAGAGTTGGGTCCCTTAAACACAAAGGAGCTTGAGCAGCTTGAGCGTCAACTTGAGTCCTCCTTGAAGCAAGTTAGGTCCACTAAGACTCAGTATATGCTGGACCAACTTTCTGACCTTCAAAATAAGGAACAAATGTTAATAGAAGCAAACAGGGATTTGTCATTGAAGTTGGATGACATTAGTTCAAGAAACCAAATTAGACAATCATGGGAAGGTGGAAACCAGGGGGGTATGGCATATGGGACCCAGCATGCTCAATCTCAAGGGTTCTTCCAGCCCTTGGATTGCAATCCCACCTTGCAAATAGGGTACTCTAATGTGGGGTCAGAGCAGATGAGTGCCACAACTCATGCCCAACAAGTCAACGGTTTCATCCCTGGATGGATGCTGTGA

>PmSEP3

ATGGGGAGGGGGAGAGTGGAGCTGAAGAGGATTGAGAACAAGATCAACAGGCAGGTGACCTTTGCGAAGCGAAGGAACGGGCTTTTGAAGAAAGCCTACGAGCTTTCCGTTCTTTGCGAGGCTGAGGTTGCTCTCATCATCTTCTCCAATAGAGGAAAGCTGTACGAGTTTTGCAGTAGCTCAAGCATGCTCAAAACTCTTGAGAGGTACCAGAAGTGCAACTATGGAGCACCAGAGACAAATGTATCTGCAAGGGAGGCCTTGGAATTGAGTAGCCAGCAGGAGTATTTGAAGCTCAAGGCACGTTATGAAGCCCTACAACGAAACCAAAGGAATCTTCTTGGAGAAGATCTTGGCCCTTTAAGCAGCAAAGAGCTTGAATCACTTGAAAGGCAGCTGGATATGTCACTGAAGCAGATCAGATCGACACGGACCCAATGCATGCTGGATCAGCTCACAGATCTTCAGCGAAAGGAGCACATGCTAAATGAAGCAAATAAGACCCTGAAACAAAGGTTGTTTGAGGGATACCACGTAAATTCACTCCAAATGAATCCAAATGCTGACGAGTACGGCCGGCAACAAGCTCAAGCTCATGGCGATGGCTTCTTCCATCCCTTAGACTGCGAGCCGACGTTACAGATTGGATACCAGAACGATCCAATATCAGTGGTCACAGCAGGTCCAAGCGTGAGTAATTACATGGCAGGATGGTTGCCATGA

>PmSEP4

ATGGGAAGAGGGAAGGTAGAGCTGAAGAGGATCGAGAACAAAATAAACCAACAGGTGACATTCGCAAAAAGACGAAATGGGTTGCTAAAGAAGGCGTACGAGCTCTCAGTTCTGTGTGATGCTGAGGTTGCTCTTATCGTCTTCTCCACCCGCGGCAAGCTCTATGAGTTCTGCAGCGGCTCTAGCATGGAGAAGACACTTGAGAGGTATCAAAGATGCAGTTATAGTGCATTGGAAGCGAGTCAACCTGCTCAGGATTCACAGAGCAGATACCAAGATTATGTGAACCTAAAAGCAAAAGTAGAGGTCCTACAGCTCACGCAGAGAAATTTTCTTGGGGAAGATTTGGGTCATTTAGGCACCAAAGAGCTCCAGCAGCTTGAGAATCAACTCGACATGTCCCTGAGGCAAATCAGGTCAACAAAGACCCAAGTTATGCATGGTCAGATTTCTGATCTTCTGAGGAAGGAACAGATGCTGCTGGAATCAAACAATGAACTGAGAAGGAAGCTGGAGGAATGCGACGCAGCCATTGAAAGATATTCATGGACAACTGAGGAGCAAAATCAAAATGTT

CCAAATAGCAGCCACCACCAGGCTGCTCAATTTGAGGGAGTCCTTGACCATTCACAATGCAACAATACATTGCAGATCGGCTACAATCCTGCTGCAGTAACGGATCATCATGAGCTACAATCCTCAACGCAAAGTCATAGTGGATTAATCTTCCCCGGGACGTGGGTGCTCTGA
