# Supplementary material for: SEP-class genes in Prunus mume and their likely role in floral organ development
Source: BMC Plant Biol. 2017 Jan 13;17:10. doi: 10.1186/s12870-016-0954-6 (PMC5234111; doi:10.1186/s12870-016-0954-6)
Supplement: Additional file 5: Table S3. — Primers used in PCR reaction. (DOCX 14 kb) [file 12870_2016_954_MOESM5_ESM.docx]

**Additional file5**

**SEP-class genes in** ***Prunus mume* and their likely role in floral organ development**

Yuzhen Zhou, Zongda Xu, Xue Yong, Sagheer Ahmad, Weiru Yang, Tangren Cheng, Jia Wang, Qixiang Zhang*

*Affiliation*: Beijing Key Laboratory of Ornamental Plants Germplasm Innovation & Molecular Breeding, National Engineering Research Center for Floriculture, Beijing Laboratory of Urban and Rural Ecological Environment, Key Laboratory of Genetics and Breeding in Forest Trees and Ornamental Plants of Ministry of Education, School of Landscape Architecture, Beijing Forestry University, Beijing, 100083, China.

******Corresponding author*

Qixiang Zhang: zqxbjfu@126.com;

**Table S3.** Primers used in PCR reaction.

| **Name** | **Sequence** | **vector** |
| --- | --- | --- |
| BK-SEP1-F | CATGGAGGCCGAATTCATGGGAAGAGGTAGAGTTGAG | pGBKT7 |
| BK-SEP1-R | GCAGGTCGACGGATCCTCAAAGCATCCACCCAGGAAT | pGBKT7 |
| BK-SEP2-F | CATGGAGGCCGAATTCATGGGGAGGGGAAGAGTGGAA | pGBKT7 |
| BK-SEP2-R | GCAGGTCGACGGATCCTCACAGCATCCATCCAGGG | pGBKT7 |
| BK-SEP3-F | CATGGAGGCCGAATTCATGGGGAGGGGGAGAGTG | pGBKT7 |
| BK-SEP3-R | GCAGGTCGACGGATCCTCATGGCAACCATCCTGC | pGBKT7 |
| BK-SEP4-F | CATGGAGGCCGAATTCATGGGAAGAGGGAAGGTAGAG | pGBKT7 |
| BK-SEP4-R | GCAGGTCGACGGATCCTCAGAGCACCCACGTCCC | pGBKT7 |
| AD-SEP1-F | GGAGGCCAGTGAATTCATGGGAAGAGGTAGAGTTGAG | pGADT7 |
| AD-SEP1-R | CGAGCTCGATGGATCCTCAAAGCATCCACCCAGGAAT | pGADT7 |
| AD-SEP2-F | GGAGGCCAGTGAATTCATGGGGAGGGGAAGAGTGGAA | pGADT7 |
| AD-SEP2-R | CGAGCTCGATGGATCCTCACAGCATCCATCCAGGG | pGADT7 |
| AD-SEP3-F | GGAGGCCAGTGAATTCATGGGGAGGGGGAGAGTG | pGADT7 |
| AD-SEP3-R | CGAGCTCGATGGATCCTCATGGCAACCATCCTGC | pGADT7 |
| AD-SEP4-F | GGAGGCCAGTGAATTCATGGGAAGAGGGAAGGTAGAG | pGADT7 |
| AD-SEP4-R | CGAGCTCGATGGATCCTCAGAGCACCCACGTCCC | pGADT7 |
