# Supplementary material for: SEP-class genes in Prunus mume and their likely role in floral organ development
Source: BMC Plant Biol. 2017 Jan 13;17:10. doi: 10.1186/s12870-016-0954-6 (PMC5234111; doi:10.1186/s12870-016-0954-6)

**Additional file6**

**SEP-class genes in** ***Prunus mume* and their likely role in floral organ development**

Yuzhen Zhou, Zongda Xu, Xue Yong, Sagheer Ahmad, Weiru Yang, Tangren Cheng, Jia Wang, Qixiang Zhang*

*Affiliation*: Beijing Key Laboratory of Ornamental Plants Germplasm Innovation & Molecular Breeding, National Engineering Research Center for Floriculture, Beijing Laboratory of Urban and Rural Ecological Environment, Key Laboratory of Genetics and Breeding in Forest Trees and Ornamental Plants of Ministry of Education, School of Landscape Architecture, Beijing Forestry University, Beijing, 100083, China.

******Corresponding author*

Qixiang Zhang: zqxbjfu@126.com;

**FigureS2.** Flower bud differentiation of *P. mume*. The flower bud development was divided into eight stages (S1-9): undifferentiation (S1), flower primordium formation (S2), sepal initiation (S3), petal initiation (S4), stamen initiation (S5), pistil initiation (S6), stamen and pistil elongation (S7), ovule development(S8), anther development (S9).The letters had different meanings. FP: Flower primordium; SeP: Sepal primordium; Se: Sepal; PeP: Petal primordium; Pe: Petal; StP: Stamen primordium; St: Stamen; CaP: Carpel primordium; Ca: Carpel; Sty: Style; An: Anther; F: Filament; Ova: Ovary; Ovu: Ovule; Po: Pollen.


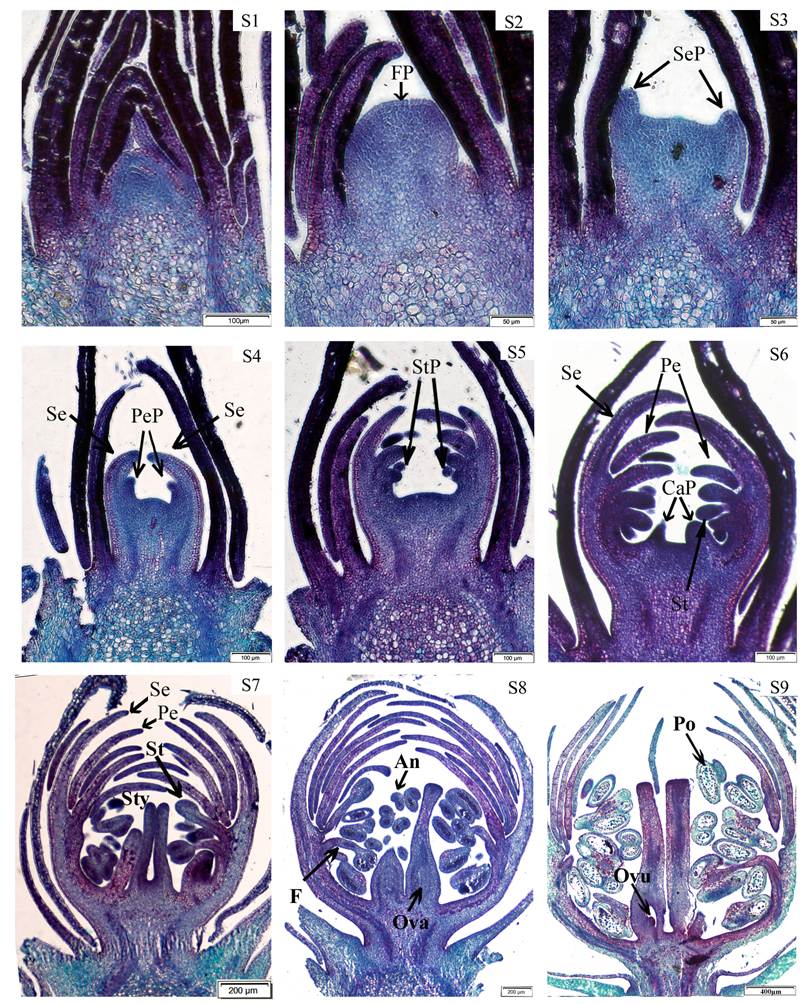

Supplement: Additional file 6: Figure S2. — Flower bud differentiation of P. mume. The flower bud development was divided into eight stages (S1-9): undifferentiation (S1), flower primordium formation (S2), sepal initiation (S3), petal initiation (S4), stamen initiation (S5), pistil initiation (S6), stamen and pistil elongation (S7), ovule development (S8), anther development (S9). The letters had different meanings. FP: Flower primordium; SeP: Sepal primordium; Se: Sepal; PeP: Petal primordium; Pe: Petal; StP: Stamen primordium; St: Stamen; CaP: Carpel primordium; Ca: Carpel; Sty: Style; An: Anther; F: Filament; Ova: Ovary; Ovu: Ovule; Po: Pollen. (DOCX 217 kb) [file 12870_2016_954_MOESM6_ESM.docx]
