# Supplementary material for: SEP-class genes in Prunus mume and their likely role in floral organ development
Source: BMC Plant Biol. 2017 Jan 13;17:10. doi: 10.1186/s12870-016-0954-6 (PMC5234111; doi:10.1186/s12870-016-0954-6)
Supplement: Additional file 7: Data S2. — The GeneBank accession numbers of genes used in alignment. (DOCX 13 kb) [file 12870_2016_954_MOESM7_ESM.docx]

**Additional file7**

**SEP-class genes in** ***Prunus mume* and their likely role in floral organ development**

Yuzhen Zhou, Zongda Xu, Xue Yong, Sagheer Ahmad, Weiru Yang, Tangren Cheng, Jia Wang, Qixiang Zhang*

*Affiliation*: Beijing Key Laboratory of Ornamental Plants Germplasm Innovation & Molecular Breeding, National Engineering Research Center for Floriculture, Beijing Laboratory of Urban and Rural Ecological Environment, Key Laboratory of Genetics and Breeding in Forest Trees and Ornamental Plants of Ministry of Education, School of Landscape Architecture, Beijing Forestry University, Beijing, 100083, China.

******Corresponding author*

Qixiang Zhang: zqxbjfu@126.com;

**Data S2**The GeneBank accession numbers of genes used in alignment are as follows: PpMADS5 (*Prunus persica*, ABO27621.1), MdMADS18 (*Malus domestica*, ADL36740.1), VvMADS4 (*Vitis vinifera*, AAM21344.1), AcSEP3 (*Actinidia chinensis*, ADU15477.1), LjSEP3 (*Lotus japonicas*, AAX13298.1), SEP3 (*Arabidopsis thaliana*, NP_564214.2), OsMADS8 (*Oryza sativa*, Q9SAR1.1), OsMADS7 (*Oryza sativa*, Q0J466.2), ZMM6 (*Zea mays*, CAD23438.1), FBP5 (*Petunia hybrid*, AAK21248.1), AcSEP1 (*Actinidia chinensis*, ADU15478.1), SEP1 (*Arabidopsis thaliana*, NP_001119230.1), SEP2 (*Arabidopsis thaliana*, NP_186880.1), VvMADS2 (*Vitis vinifera*, NP_001268109.1), MdMADS1 (*Malus domestica*, AAC25922.1), FaMADS1 (*Fragaria ananassa*, AEF59025.1) , PpMADS7 (*Prunus persica*, ABO27622.1), MdMADS3 (*Malus domestica*, AAD51422.1), AcSEP4 (*Actinidia chinensis*, ADU15479.1), FBP9(*Petunia hybrid*, AAK21249.1), FBP23 (*Petunia hybrid*, AAK21254.1), SEP4 (*Arabidopsis thaliana*, NP_178466.1), MdMADS4 (*Malus domestica*, AAD51423.1).
